# The impact of antimicrobial allergy labels on antimicrobial usage in cancer patients

**DOI:** 10.1186/s13756-015-0063-6

**Published:** 2015-06-01

**Authors:** Jason A. Trubiano, Vivian K. Leung, Man Y. Chu, Leon J. Worth, Monica A. Slavin, Karin A. Thursky

**Affiliations:** Department of Infectious Diseases, Peter MacCallum Cancer Centre, East Melbourne, VIC Australia; Department of Infectious Diseases, Austin Health, East Melbourne, VIC Australia; Victorian Infectious Diseases Service, Peter Doherty Institute, Melbourne, Australia

**Keywords:** Antibiotic allergy, Stewardship, Cancer, Antimicrobial resistance

## Abstract

**Background:**

Antibiotic allergy labels are associated with sub-optimal prescribing patterns and poorer clinical outcomes in non-cancer populations, but the effect of labelling on antimicrobial usage in patients with cancer is unknown.

**Findings:**

A retrospective review of hospitalized patients admitted to the Peter MacCallum Cancer Centre (2010-2012) identified 23 % of cancer patients (n = 198) with an antimicrobial allergy label (AA). Comparison of those with an antimicrobial allergy label to those without demonstrated increased antibiotic use per admission (3 vs. 2, p = 0.01), increased fluoroquinolone use (11 % vs. 6 %, p < 0.05), increased antibiotic course duration (15 vs. 13 days, p = 0.09), higher readmission rates (53 % vs. 28 %, p < 0.001) and poorer concordance with prescribing guidelines (47 % vs. 91 %, p < 0.001). Patients in the AA group on multivariate analysis had a higher number of antibiotics employed, longer duration of antibiotic therapy and higher rate of readmission.

**Conclusions:**

Antimicrobial usage, including the use of restricted antibiotics, is higher in patients with cancer. Antibiotic de-labelling strategies in cancer patients must be evaluated to aid antimicrobial stewardship initiatives.

## Background

Despite 10-20 % of patients reporting an antimicrobial allergy, less than 1 % of patients test positive on skin-prick-testing/intradermal testing (SPT/IDT) [1–3]. Recent studies in mixed populations demonstrate increased antimicrobial use, poorer patient outcomes and development of microbiological resistance in those with an antibiotic allergy label [3–5]. However, the impact of antimicrobial allergy labels on cancer patients and antimicrobial usage has not yet been defined. The objectives of our study were to: (i) determine the prevalence of antimicrobial allergy labels in patients with cancer, (ii) provide a description of reported antibiotic allergies and re-challenge history, and (iii) to describe the impacts of an antimicrobial allergy label on antimicrobial choice, usage and clinical outcomes.

## Methods

### Study design, setting and population

Peter MacCallum Cancer Centre (PMCC) is a tertiary referral center treating all cancer patients. This was a single center retrospective review of oncology and hematology patients admitted to PMCC between June 2010 and July 2012 with a coded infective diagnosis according to the International Classification of Disease-10-AM. Study inclusion criteria were: (i) coded infective diagnosis, (ii) receipt of an antimicrobial agent for treatment of infection, and (iii) inpatient admission > 24 h. The study was approved by the PMCC ethics- committee.

Using medical and pharmacy records, patients were divided into two study groups: (i) those with an antimicrobial allergy label (AA), reporting an allergy or adverse drug reaction to an antimicrobial and (ii) those without no antimicrobial allergy label (NAA). The AA group included patients with Type A and/or Type B antimicrobial adverse drug reactions (see Definitions below). The pharmacy records and allergy particulars are updated by a dedicated ward pharmacist for all hospital admissions.

For all patients, demographics characteristics, infective diagnosis, age-adjusted Charlson Comorbidity Index (CCI), [6] mental health and malignancy history was recorded. Antimicrobial usage for each antibiotic administered during initial and subsequent admissions during the study period was recorded: agent(s), doses, frequency, route of administration and duration. Patient re-admission rate and outcomes (inpatient 30-day, and 60-day mortality) were captured.

For the AA patients additional information obtained included; allergy description, reporting clinician, re-challenge history during study period, de-sensitization history during study period and allergy/immunologist specialist referral. Patients were excluded if antibiotic administration records were incomplete.

### Definitions

AA labels were classified as either Type A or Type B based upon the following criteria:

- Type A: Non-immune mediated adverse drug reactions consistent with a known drug side effect (e.g. gastrointestinal upset).
- Type B: Reactions consistent with an IgE-mediated (e.g. angioedema, anaphylaxis or urticaria) OR T-cell mediated (e.g. serum sickness, antibiotic induced hemolytic anemia, maculopapular eruption or Severe Cutaneous Adverse Reaction) reaction.

For the purposes of this study, concordance with prescribing guidelines was determined by comparing antibiotic choice with first-line recommendations provided by the Australian Therapeutic Guidelines (Antibiotic) and national guidelines for management of neutropenic fever [7, 8]. An antibiotic course was defined as > 24 h of antibiotic therapy targeting an infective diagnosis. An antimicrobial allergy manifestation was defined as any reported allergy or adverse drug reaction (Type A or B) to an antimicrobial, listed in the allergy box of medical record.

### Statistical analysis

Statistical analyses, including multivariate analysis, were performed using Stata 12.0 (Statacorp, Texas). Clinical outcomes were compared between the AA and NAA groups. Categorical variables were compared using the chi-squared test and continuous variables compared using the Wilcoxon rank sum test. A *P*-value of <0.05 was deemed statistically significant.

## Results

### Patient demographics

198 patients with at least one infective episode were identified during the study period. Of these, 45 (23 %) had an antimicrobial allergy label (AA) and 153 (77 %) had no label (NAA). The groups were comparable with respect to age, sex, Charlson Comorbidity Index, mental health history, ICU-admission rate and mortality /30-day/60-day (Table 1).

**Table 1:** Characteristics of AA and NAA groups

Patient characteristics	No label	Label	p-value
	(N = 153)	(N = 45)	
	n (%)	n (%)	
Age (years)			
Median (range)	65 (22-92)	64 (22-90)	0.48
Charlson Comorbidity Index^a^			
Median (range)	4 (2-12)	4 (2-10)	0.36
Sex			
Male	86 (56)	20 (44)	0.16
Mental health history^b^			
Yes	24 (16)	7 (16)	0.98
Treating unit			
Hematology	93 (61)	27 (60)	0.85
Medical Oncology	44 (29)	12(27)	
Surgical Oncology	30 (20)	8 (18)	0.51
Malignancy type			
Leukemia	29 (19)	13 (29)	
Lymphoma	60 (39)	17 (38)	
Solid Malignancy	27 (18)	6 (13)	
Multiple myeloma			
Inpatient surgery			
Yes	26 (17)	9 (20)	0.87
ICU admission			
Yes	39 (26)	12 (27)	0.87
Infectious episodes			
Febrile neutropenia	70 (21)	24 (24)	0.54
Gram negative bacteremia	31 (9)	12 (12)	
Gram positive bacteremia	25 (8)	6 (6)	
Polymicrobial bacteremia	11 (3)	2 (2)	
Skin and soft tissue infection	16 (5)	10 (10)	
Pneumonia	19 (6)	8 (8)	
Invasive fungal infection	18 (6)	4 (4)	
Intra-abdominal collection	13 (4)	3 (3)	
Mortality (all-cause)			
30 day	6 (3.9)	1(2.2)	0.59
60 day	10 (6.5)	2 (4.5)	0.89
Length of Stay			
Median (range)	19 (2-151)	23 (3-157)	0.4618
Readmissions			
Median (range)	2 (0-11)	3.5 (1-17)	0.02
Readmission with an infectious diagnosis			
Yes	42 (27.5)	24 (53.3)	0.01

^a^ Age-adjusted Charlson Comorbidity Index [6]

^b^ Mental Health History: Recorded from patient history

### Allergy labels & manifestations

In the AA patients, 62 allergy manifestations were recorded. Ten of the 62 allergy manifestations (16 %) were Type A adverse drug reactions (gastrointestinal 5; other 5). Of the Type B allergies (52/62; 84 %), IgE and T-cell mediated manifestations were reported in 8/52 (15 %) and 44/52 (85 %) respectively. Antibiotic allergies were most commonly attributed to beta-lactams (65 %), followed by sulfonamides (11 %), glycopeptides (6 %), fluroquinolones (5 %), macrolides (5 %) and tetracyclines (2 %). More specifically, the causal antibiotic(s) included penicillin/aminopenicillins 23/62 (46 %), piperacillin-tazobactam 9/62 (14 %), trimethoprim/sulfamethoxazole 7/62 (11 %), vancomycin 4/62 (7 %), flucloxacillin 3/62 (5 %), ciprofloxacin 3/62 (5 %), cephalexin 2/62 (3 %), ceftazidimine 1/62 (2 %), cefepime 1/62 (2 %), cefuroxime 1/62 (2), dapsone 2/62 (3 %), metronidazole 2/62 (3 %), roxithromycin 2/62 (3 %), and doxycycline 1 (2 %).

Re-challenge with the offending antimicrobial was more likely if the allergy label was non-IgE mediated compared with IgE mediated (p = 0.024). Six of 44 patients with a rash (14 %) and 3/10 (30 %) with a Type A history were re-challenged. No formal SPT was recorded for the AA group. The health professional reporting the antimicrobial allergy was unknown in 37/62 (60 %), physician in 19/62 (30 %), pharmacist 3/62 (5 %), surgeon 2/62 (3 %) and general practitioner 1/62 (2 %).

### Antimicrobial usage & multivariate analysis

**Fig. 1:**
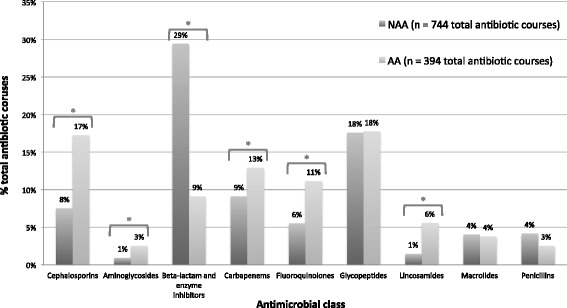
The percentage of total antibiotic courses (all admissions during study period) per antimicrobial class for antimicrobial allergy (AA) and no antimicrobial allergy (NAA) groups. Abbreviations: AA, antimicrobial allergy group; NAA, no antimicrobial allergy group. *P value < 0.05

The median number of antibiotics used per admission was significantly higher in the AA group compared with the NAA group (3 vs. 2, p = 0.01), with a trend toward longer antibiotic duration (15 days vs. 13 days, p =0.09). Concordance with 1^st^ line therapy occurred less frequently in the AA group (21/45; 47 % vs. 139/153; 91 %, p < 0.001). There was no difference in 30-day or 60-day mortality and LOS in the two groups. The median number of readmissions and/or readmissions with an infectious disease diagnosis requiring antimicrobial therapy was higher for the AA group (Table 1). For both first admission and re-admissions, ciprofloxacin (11.7 % vs. 4.7 %, p < 0.001), meropenem (12.1 % vs. 9.1 %, p = 0.21) and cefepime (9.3 % vs. 1.2 %, p < 0.001) use as percentage of total antibiotic courses was higher in AA group compared to NAA group. There was no difference in vancomycin use between the 2 groups (15 % vs. 15 %, p = 0.9). The percentage of total antibiotic courses per antimicrobial class for the AA and NAA groups is demonstrated in Fig. 1.

On multivariate logistic regression adjusting for age group, inpatient surgery, sex, ICU admission and LOS there was a higher readmission rate with an infective diagnosis (OR 3.27 95%CI 1.55-6.88, p = 0.002) and overall readmission rate (OR 1.99, 95%CI 0.95-4.15, p = 0.069) in the AA group compared with NAA group. The number of antibiotics employed (regression coefficient 0.57, 95%CI 0.09-1.06, p = 0.021) and duration of antibiotic therapy (regression coefficient 6.70, 95%CI 0.84-12.5, p = 0.03) was higher in the AA group.

## Discussion

In a cohort of hospitalized patients with cancer and an inter-current infection, we identified on multivariate analysis significant impacts of antimicrobial allergy labelling, including (i) increased total number of administered antibiotics, (ii) increased antibiotic duration and (iii) higher re-admission rates. Previously, the prevalence of allergy labelling in non-surgical patients has been estimated to be 11 %, with increased antibiotic usage, length-of-stay and mortality noted in the allergy label group [4]. Penicillin allergy labels in mixed patient populations have been associated with increased antimicrobial usage [5]. However, our study is the first to identify a high rate of antimicrobial allergy labels (23 %) in a cancer patient cohort with demonstrable effects on antimicrobial prescribing.

Increasing rates of multi-drug resistant organisms are observed amongst cancer patients, along with increasing fluoroquinolone and carbapenem use [9–11]. We demonstrated during the study period an increase in fluoroquinolone and carbapenem use in our AA group. Improving pathways to ‘de-labeling’ may reduce the unwarranted use of these agents [3, 12]. The high rate of rash manifestations (71 %), yet low rate of direct re-challenge in this population suggests a need for more comprehensive evaluation. For many ‘rash’ manifestations, the reported exanthem is frequently mild, not directly related to antimicrobial agents [13]. Enhanced education and introduction of programs to assess allergy status will reduce the number of antimicrobial allergy labels and may allow these patients to be safely re-challenged. Remaining IgE-mediated manifestations should be further investigated with SPT/IDT as part of a de-labeling program [3, 14, 15].

The main limitation of this study is the retrospective study design that restricted the ability to clarify the nature of specific antimicrobial allergy labels. However, dedicated ward pharmacist roles during the study period enabled capture of data upon hospital admission, and we believe our data to be valid. Other limitations include small study numbers and heterogeneity of cancer populations. The use of ICD-10-AM coding data to identify patients is likely to have underestimated the number of infectious episodes. However, this would be applicable to both of our study groups and therefore unlikely to affect the studied outcomes.

## Conclusion

In summary, almost one quarter of patients admitted to our centre with cancer and an infection had an antimicrobial allergy label. Antibiotic administration rates were higher and duration longer in the AA group. The potential benefits of antimicrobial allergy ‘de-labeling’ strategies must be evaluated prospectively in at-risk cancer populations to aid antimicrobial stewardship programs and prevent resistance generation.

## Abbreviations

ICU: Intensive Care Unit
d: day
R: range
